# Modulatory effect of *Gracilaria gracilis* on European seabass gut microbiota community and its functionality

**DOI:** 10.1038/s41598-022-17891-9

**Published:** 2022-09-01

**Authors:** Ana Teresa Gonçalves, Marco Simões, Cátia Costa, Ricardo Passos, Teresa Baptista

**Affiliations:** 1 MARE-Marine and Environmental Sciences Centre, Polytechnic of Leiria, Edifício CETEMARES, Av. Porto de Pesca, 2520-620 Peniche, Portugal; 2 School of Tourism and Maritime Technology, Polytechnic of Leiria, Campus 4, Rua do Conhecimento, nº 4, 2520-641 Peniche, Portugal; 3 GreenCoLab-Associação Oceano Verde, University of Algarve, Campus of Gambelas, Bldg7, Faro, Portugal

**Keywords:** Biotechnology, Genetics

## Abstract

Seaweeds are an important source of nutrients and bioactive compounds and have a high potential as health boosters in aquaculture. This study evaluated the effect of dietary inclusion of *Gracilaria gracilis* biomass or its extract on the European seabass (*Dicentrarchus labrax*) gut microbial community. Juvenile fish were fed a commercial-like diet with 2.5% or 5% seaweed biomass or 0.35% seaweed extract for 47 days. The gut microbiome was assessed by 16S rRNA amplicon sequencing, and its diversity was not altered by the seaweed supplementation. However, a reduction in Proteobacteria abundance was observed. Random forest analysis highlighted the genera *Photobacterium*, *Staphylococcus*, *Acinetobacter*, *Micrococcus* and *Sphingomonas,* and their abundances were reduced when fish were fed diets with algae. SparCC correlation network analysis suggested several mutualistic and other antagonistic relationships that could be related to the predicted altered functions. These pathways were mainly related to the metabolism and biosynthesis of protective compounds such as ectoine and were upregulated in fish fed diets supplemented with algae. This study shows the beneficial potential of *Gracilaria* as a functional ingredient through the modulation of the complex microbial network towards fish health improvement.

## Introduction

The vertebrate gastrointestinal tract (GIT) is a crowded and complex ecosystem inhabited by microbial communities of bacteria, fungi and archaea that over the last decades has demonstrated to be particularly important in the health and welfare of the hosts^1,2^.

Healthy conditions contribute to a balanced and diversified gut microbiota, preventing its dysregulation or dysbiosis, and promoting beneficial symbiotic interaction with the host^3^. In this symbiosis, while the host provides a good environment and nutrient supply, gut microbiota plays a critical role in nutrient digestion and absorption^4,5^, appetite regulation^6^, immune response^7^, protection from pathogenic microorganisms^8^ and gene expression regulation^9^. However, gut microbiomes are shaped by several factors, including trophic level^10^, environmental conditions^11^ and feed source^12,13^.

Bacteria communities´ density and composition vary along the GIT depending on the physical and chemical conditions^14,15^. Generally, bacterial density increases progressively along with the GIT, being the intestine the region with high alpha-diversity indices^16,17^. In addition, some bacteria species are present in all GIT, varying their abundance, while others are specific to some GIT regions^18,19^. These variations are related to pH^19^, protease activity^20^, amount and type of available nutrients^18^, and adhesion capacity of bacteria groups to the epithelial cells or mucus^21^ among other factors. It is important to understand the dynamics of the microbial community in the gut in order to relate with the metabolic and physiological impacts of nutritional and health alterations in fish^12,22^.

In livestock productions such as aquaculture, a balanced microbial community gains particular importance^23^ due to the captive breeding and rearing conditions^24^. High stocking densities and high levels of stress can lead to disease spreading/outbreaks^25^. European seabass (*Dicentrarchus labrax*) is one of the most relevant produced species in southern Europe, and its production is strongly affected by bacterial diseases, mainly photobacteriosis, vibriosis and tenacibaculosis^26^. Regarding the photobacteriosis, the responsible pathogenic agent is *Photobacterium damselae* subsp. *piscicida*. This bacterium is considered by worldwide fish farmers as one of the most dangerous microorganisms due to its ubiquitous distribution, high mortality rate and large fish species spectrum^27^ including *D. labrax*^28,29^. This disease is characterized by acute septicaemia in young fish and granulomatous lesions in adults^27^, reaching high mortality rates of 60–80% in European seabass farms^30,31^.

Outbreaks of these and other diseases are one of the major problems that aquaculture has been facing that have been fought and prevented through antibiotics^32^. However, the use of antibiotics not only promotes the resistance acquisition in the pathogens^33,34^ but also diminishes the gut-microbiota diversity in aquaculture fish^35,36^, increasing disease susceptibility^37,38^. However, it is known that dietary modulation^39,40^ can increased food uptake and can also modulate the gut microbiota composition^14^. Here, the use of functional feeds is foreseen as a strategy to enhance fish health in aquaculture.

Functional feeds are defined as feeds enriched with selected ingredients, that provide benefits to the fish’s health status^41^. Algae have been considered a good source of ingredients to add to aquafeeds^42^. Due to their richness in bioactive compounds, seaweeds have a very high potential regarding antibacterial, antifungal and antioxidant capabilities^43^, among others. Thus, the interest in using these organisms as immunomodulators is increasing, with evidence of immunostimulatory effects when applied to aquaculture feeds^44^. In particular, seaweeds from the genus *Gracilaria* (Gracilariaceae, Rhodophyta) have demonstrated to be a good source of bioactive compounds with antioxidant, radical scavenging and antimicrobial activities^45–48^, and also as aquafeed supplement^49,50^ for health improvement. Several compounds related to antimicrobial activity have been found in *Gracilaria gracilis*, such as R-phycoerythrin, arachidonic acid, proteins, and phenols^45,48^. More, the *G. gracilis* extracts’ ability to inhibit *Vibrio fischeri*^48^ and *Photobacterium damselae* subsp. *damselae* bacteria growth has been reported^51^, supporting its potential as a nutritional strategy in aquaculture.

Polysaccharides and other bioactive compounds, plentiful in seaweeds, have been described as prebiotics^52^. These compounds modulate fish intestinal microbiota stimulating the proliferation of beneficial bacterial populations, with positive physiological consequences for the host. This is commonly associated with the production of beneficial compounds such as short-chain fatty acids^52^. Despite all these potential properties, the inclusion of algae in percentages above 10% has shown negative effects on fish growth and other zootechnical parameters^53,54^. This might be due to the presence of anti-nutritional factors (ANF) that can interfere with the digestive process, such as lectins and protease inhibitors^55,56^. However, aquafeeds enriched with an algae percentage below 10%, brought advantages to the growth, nutrient utilization, feed efficiency and disease resistance^57–59^. Also, algae dietary inclusion modulated fish gut microbiota, increasing diversity^57,60^, resulting in healthier and more resistant fish^59^.

Recently, it has been reported by the same research group of the present work that European seabass fed with *Gracilaria gracilis* supplemented diets obtain a general health improvement^49^. Following this study, it was questioned a possible role of microbiota modulation that resulted in this positive output. Therefore, as a continuation of the referred study, this work aimed to assess the effect of the dietary supplementation with the seaweed, *Gracilaria gracilis*, biomass and its extract in *Dicentrarchus labrax* gut-microbiota through high-throughput sequencing technology. It was also aimed to evaluate the possible modulation of functional processes in the microbial community, using functional prediction tools.

## Results

### Fish performance and mortality

Seabass growth performance, feed conversion ratio and overall health improvement have been previously reported^49^. Briefly, weight gained and feed conversion ratio (FCR) by the end of the trial were not significantly different among groups, however fish fed diet with 5% seaweed inclusion tend to gain more weight and to convert better. There was no mortality throughout the trial all fish presented normal behaviour and reaction to feeding moments.

### 16S rRNA amplicon sequencing output and microbial community analysis

**Figure 1:**
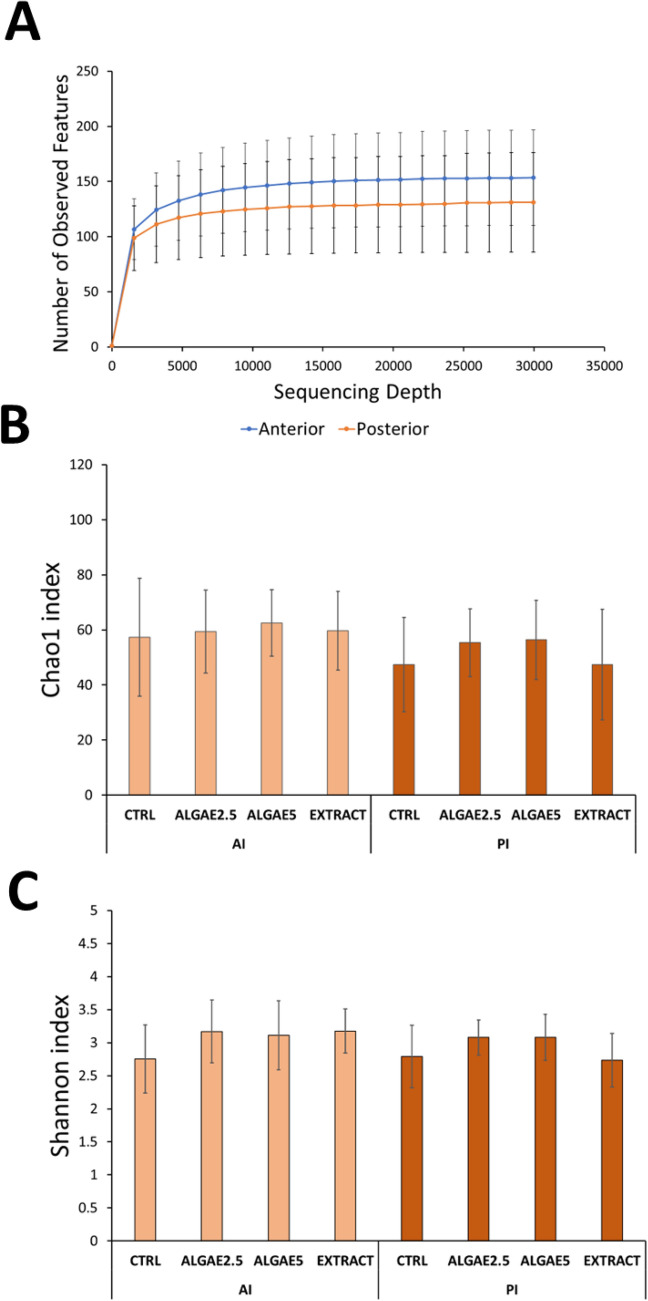
Rarefaction curve (**A**), chao1 richness index (**B**) and Shannon diversity index (**C**) of European seabass gut microbial communities for anterior and posterior intestine. Rarefaction curve indicates number of observed features for anterior (blue) and posterior (orange) intestine depending on sequencing depth. Fish were fed a basal diet with no supplement (CTRL) or supplemented with *Gracilaria gracilis* powdered biomass at 2.5% (ALGAE2.5), at 5% (ALGAE5) or with the seaweed extract at 0.35% inclusion rate (EXTRACT). No significance differences were observed; dots (in **A)** and bars (in **B** and **C**) indicate mean value of the group and error lines indicate ± SD; AI and PI stand for anterior and posterior intestine respectively (Kruskall–Wallis, P > 0.05).

**Figure 2:**
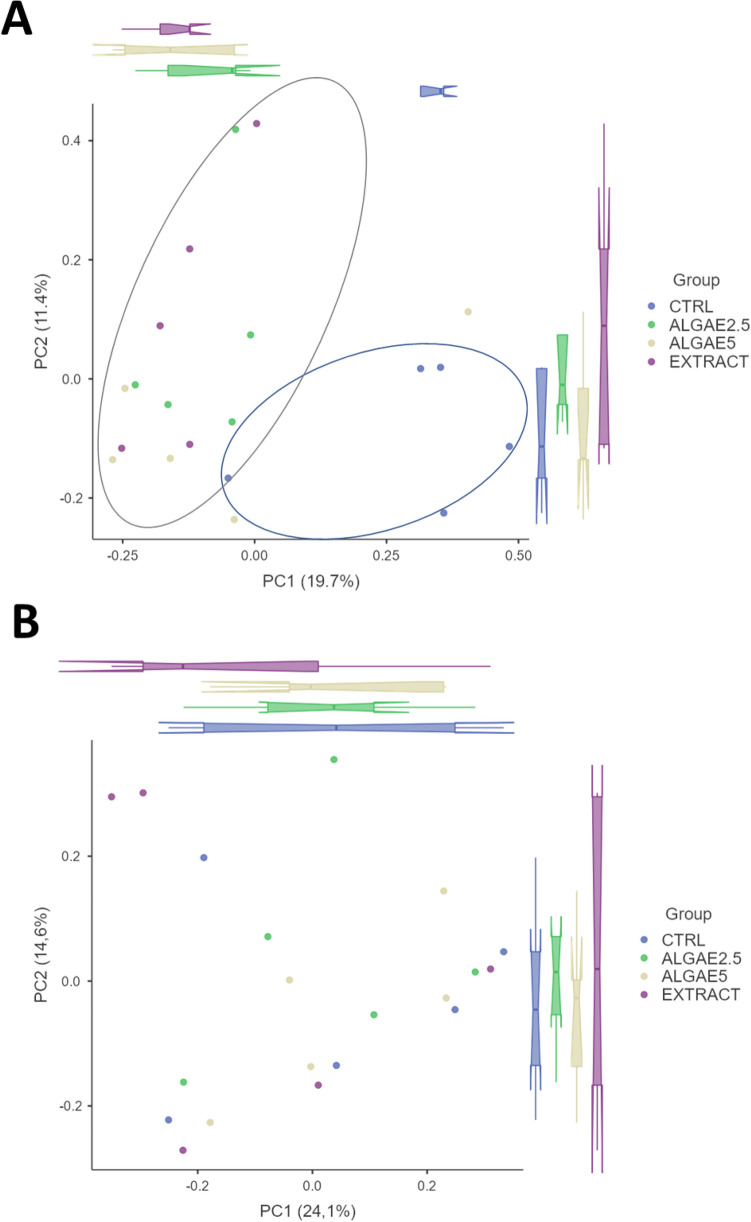
Principal coordinate analysis (PCoA) of Bray–Curtis dissimilarities observed in seabass anterior (**A**) and posterior intestine (**B**) microbial communities. Box plots represent the average coordinate for each group in the correspondent axis (i.e., PC1 or PC2). Ellipses indicate a significant separation between CTRL group (right ellipse) and other groups (left ellipse) based on PERMANOVA (P = 0.003). Fish were fed a basal diet with no supplement (CTRL) or supplemented with *Gracilaria gracilis* powdered biomass at 2.5% (ALGAE2.5), at 5% (ALGAE5) or with the seaweed extract at 0.35% inclusion rate (EXTRACT).

A total of 5,177,608 raw reads were quality filtered and merged into 1,742,241 sequences (Supplementary Table 1) in an average of 35.4% non-chimeric high-quality sequences. From this, a total of 3315 ASVs were clustered, however, only 571 had more than 2 counts and were used in further downstream analysis. After rarefaction all samples reached a plateau (Fig. 1A) of observed features, indicating proper sequencing depth. Microbial communities’ richness (i.e., Chao1 index) was not modulated by diets (Fig. 1B; P > 0.05) and neither was the Shannon diversity index (Fig. 1C; P > 0.05). On the other hand, the gut community’s beta diversity based on the Bray–Curtis dissimilarities indicated that in the anterior intestine the structure of the gut microbial communities is modulated by the inclusion of *Gracilaria gracilis* biomass or extract (Fig. 2A; P < 0.02). However, the bacterial communities in the posterior intestine do not seem to be modulated by diets (Fig. 2B).

**Figure 3:**
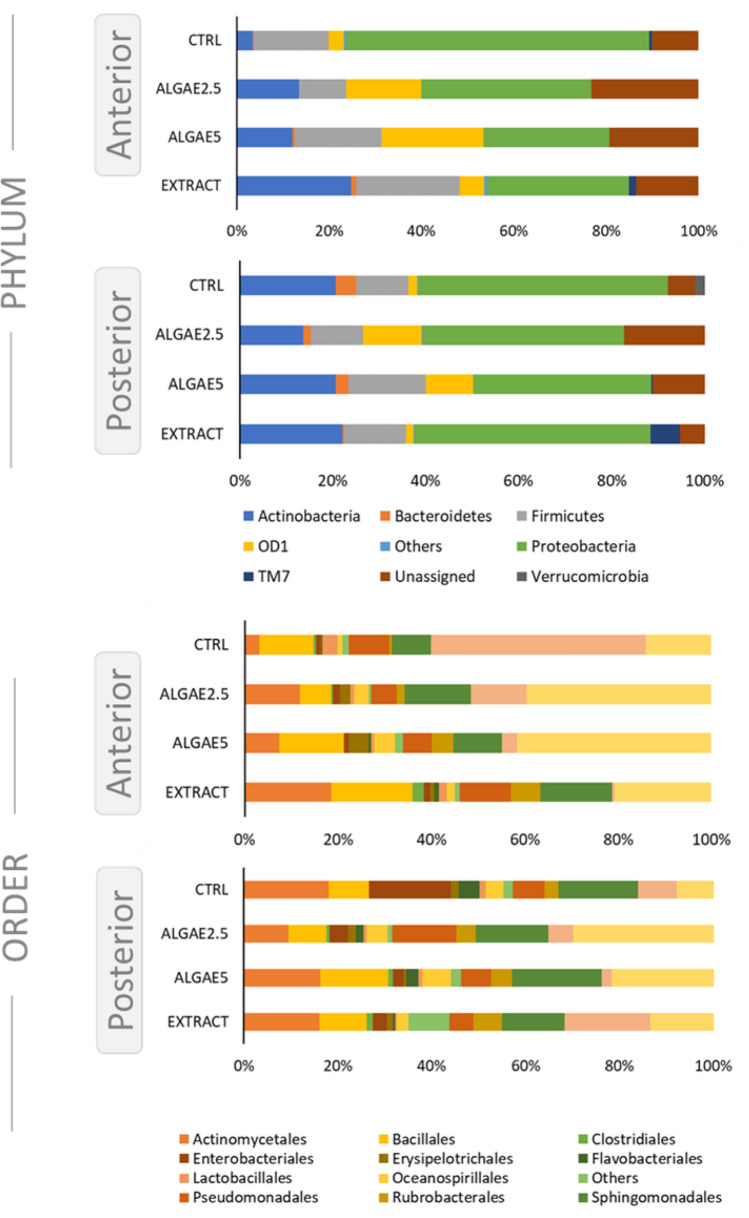
Relative phyla (upper charts) and order (lower charts) abundance of European seabass gut microbial communities. Fish were fed a basal diet with no supplement (CTRL) or supplemented with *Gracilaria gracilis* powdered biomass at 2.5% (ALGAE2.5), at 5% (ALGAE5) or with the seaweed extract at 0.35% inclusion rate (EXTRACT).

The most dominant phylum in both intestine sections was Proteobacteria, followed by Actinobacteria, Firmicutes and OD1 (Fig. 3). In the anterior intestine, a clear reduction in Proteobacteria abundance is observed when fish were fed algae or extract supplemented diet, whereas Actinobacteria and OD1 increase, except in the group fed the extract for the latter. In the posterior intestine, changes are not so evident, but abundance modulation was also observed. Regarding the order, abundances were modulated differently between intestine sections. In the anterior intestine Actinomycetales and Sphingomonadales abundance increased in group ALGAE2.5 and EXTRACT, whereas Bacillales abundance increased only in the EXTRACT group. However, Vibrionales abundance was strongly reduced in all groups with ALGAE or Algae EXTRACT dietary inclusion. On the other hand, in the posterior section, the major differences were observed in the abundance of Enterobacteriales which was reduced in all Algae related groups. In this intestinal section, Vibrionales abundance was higher in the group fed with a diet supplemented with the EXTRACT.

**Figure 4:**
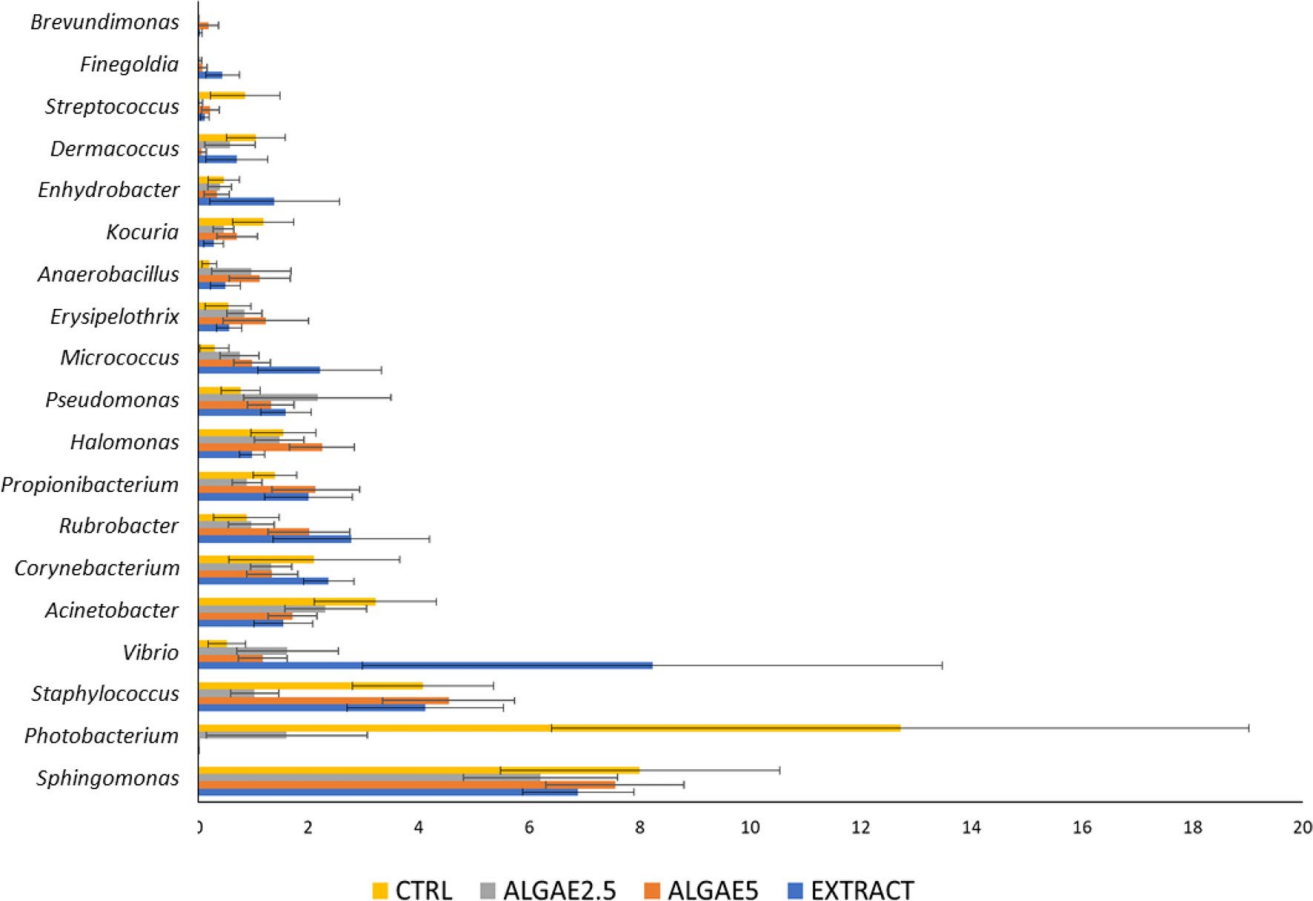
Relative abundance of most abundant genera in European seabass gut microbial communities when fed the experimental diets, and these were a basal diet with no supplement (CTRL) or supplemented with *Gracilaria gracilis* powdered biomass at 2.5% (ALGAE2.5), at 5% (ALGAE5) or with the seaweed extract at 0.35% inclusion rate (EXTRACT). Bars and errors indicate mean ± SD of differentially abundant genera (ANOVA, P < 0.05).

The most abundant and differentially modulated genus were *Sphingomonas*, followed by *Photobacterium, Staphylococcus* and *Vibrio* (Fig. 4). In the first three, abundances in the control group (CTRL) gut community are higher, especially in the case of *Photobacterium*. However, the highest abundance of *Vibrio* was observed in the communities of fish fed diet supplemented with seaweed EXTRACT.

**Figure 5:**
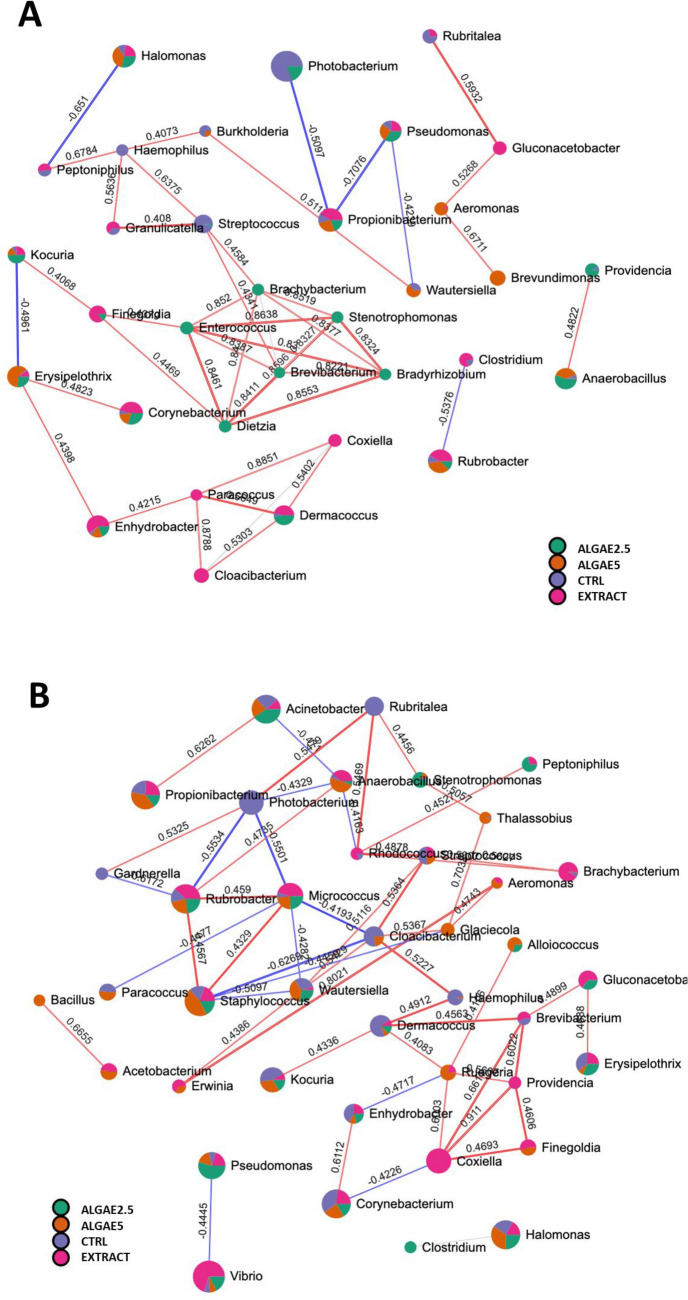
Correlation network analysis between microbial community of European seabass anterior (**A**) and posterior (**B**) intestine at genus level, on based on SparCC algorithm. Network nodes pie charts represent genus abundance per dietary group, and edges represent correlation between genera pairs where blue and red edges indicate negative and positive correlation respectively. Significant correlation threshold was set to 0.4 with P < 0.05. Fish were fed a basal diet with no supplement (CTRL) or supplemented with *Gracilaria gracilis* powdered biomass at 2.5% (ALGAE2.5), at 5% (ALGAE5) or with the seaweed extract at 0.35% inclusion rate (EXTRACT).

Correlation network highlighted several potential relationships between genera based on their abundance in each group (Fig. 5). In anterior intestine (Fig. 5A) it is noticeable a central cluster of genera positively correlated and with higher abundance in ALGAE2.5 group, and this includes the genera *Enterococcus*, *Brachybacterium*, *Stenotrophomonas*, *Bradyrhizobium*, *Brevibacterium* and *Dietzia*. *Photobacterium* genus highlights with higher abundance in CTRL group and negatively correlated with *Propionibacterium*, and a smaller cluster is visible with genera with higher abundance in the EXTRACT group that includes the *Enhydrobacter*, *Cloacibacterium*, *Paracoccus*, *Coxiella* and *Dermacoccus*, all positively correlated among them. In the posterior section, network is more diffused, and a minor cluster is evidenced where genera with higher abundance in CTRL group are positively correlated, and this includes *Cloacibacterium*, *Streptococcus*, *Haemophilus*, *Dermacoccus* and *Kokuria*, whereas another smaller cluster evidence genera with higher abundance in EXTRACT group such as *Providencia*, *Finegoldia*, *Brevibacterium*, *Coxiella* and *Gluconacetoba*.

**Figure 6:**
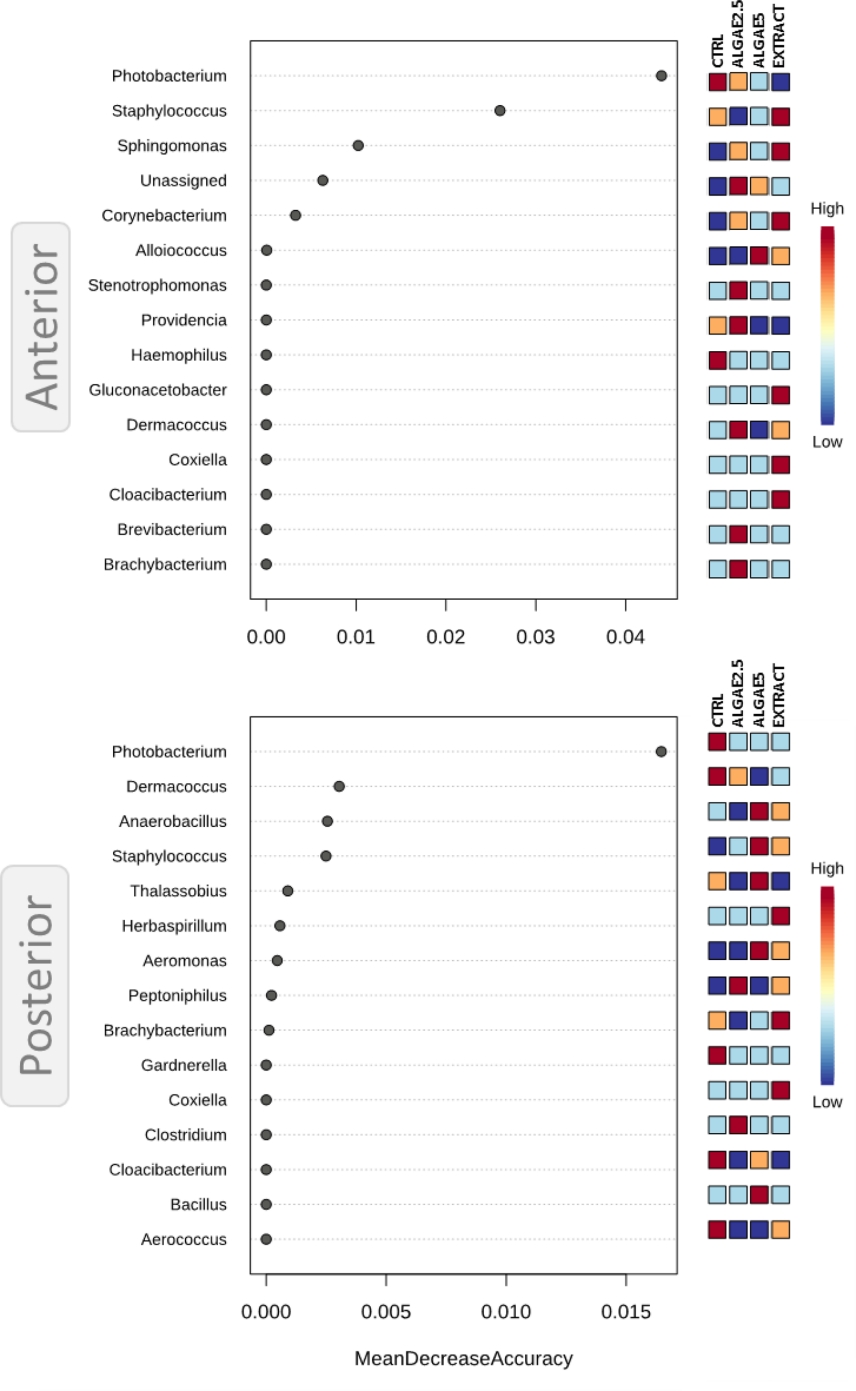
Identification of significant features on the European seabass gut microbial community when fed different diets. Analysis was based on Random Forest analysis and plot (left side) represents important features for the respective intestine section, whereas mini heatmap (right side) shows the pattern of change across different groups. Fish were fed a basal diet with no supplement (CTRL) or supplemented with *Gracilaria gracilis* powdered biomass at 2.5% (ALGAE2.5), at 5% (ALGAE5) or with the seaweed extract at 0.35% inclusion rate (EXTRACT).

Random Forest analysis unravelled the most important genera within the groups’ intestinal microbial communities. This assessment is based not on the abundance value but on the magnitude of the modulation of the abundance depending on treatments. Thus, in both intestinal sections the genus *Photobacterium* was highlighted as the most important feature for these communities (Fig. 6A,B). This genus abundance was significantly higher in CTRL group with a negative modulation exerted by diets supplemented with ALGAE biomass and EXTRACT (P < 0.03 and P < 0.0001 in anterior and posterior intestine respectively). In the anterior intestine community, *Staphylococcus* and *Sphingomonas* also presented a relevant role (Fig. 6A) mainly due to their abundance increase in the group fed with EXTRACT, whereas in posterior intestine community *Dermacoccus*, *Anaerobacillus* and *Staphylococcus* were the most relevant features of the community (Fig. 6B). Here only *Dermacoccus* had a reduction of abundance when fish were fed algae related diets, since the latter two genera presented higher abundances when fish were fed 5% seaweed supplemented diets.

### Microbiome functional prediction

Microbiome functional profile prediction was performed based on metabolic pathways, and a hierarchical clustering revealed that the microbiomes functions of fish fed CTRL or ALGAE related diets tend to be different, mainly in the anterior intestine (Supplementary Fig. 2A). Here, communities of fish fed with CTRL diet cluster closely (orange samples in heatmap), and except for two samples of fish fed algae supplemented diet all algae related samples clustered (blue and green samples on heatmap) together (regardless if supplementation was with seaweed biomass or extract). However, in the posterior intestine (Supplementary Fig. 2B) this differentiation is not as evident, and samples present more similar functional patterns (samples with mixed clustering).

**Figure 7:**
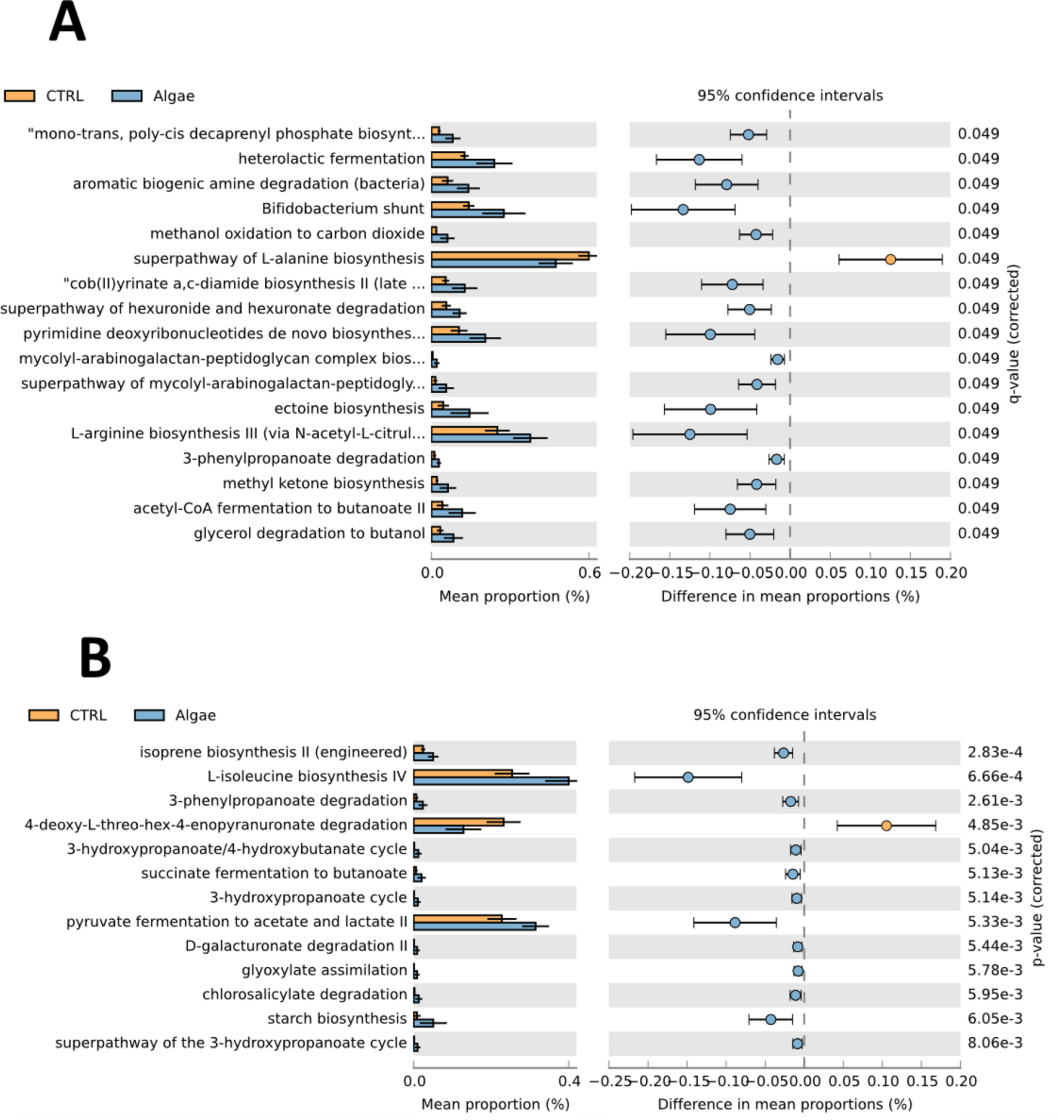
Modulation of the most significant microbiome predicted metabolic pathways in anterior (**A**) and posterior (**B**) intestine. Extended error bars indicate mean proportion of the metabolic pathway in CTRL group (orange) and groups fed with diet supplemented with algae (blue), while errors (right side) are 95% confidence interval of the difference of mean proportions in CTRL and algae groups.

When evaluating the metabolic pathways abundance (i.e., predicted enrichment) in the anterior intestine (Fig. 7A) 16 out of 17 pathways are enhanced in the microbiome of fish fed either 2.5% or 5% ALGAE biomass supplemented diets. The only pathway that is less enriched in the ALGAE group is the super pathway of l-alanine biosynthesis, whereas several present strong enhancement in this group (e.g. heterolactic fermentation, Bifidobacterium shunt, cob (II)yrinate a,c-diamide biosynthesis II, ectoine biosynthesis, among others). In the posterior intestine (Fig. 7B) less predictive enrichment was observed, however, 12 out of 13 differentially enriched pathways were enhanced in ALGAE groups compared to CTRL. Here we highlight l-isoleucine biosynthesis V, succinate fermentation to butanoate (not the highest mean proportion but with high significance), the pyruvate fermentation to acetate and lactate II and the 3-phenylpropanoate degradation.

## Discussion

Seaweed’s potential for health management has been highlighted over the last years. The antioxidant, immunostimulant, and overall health-enhancing effects exerted by seaweeds have been pointed to as the main reasons for its health-promoting qualities^61,62^. Although a large amount of the available reports refer to the effects on human health, several studies have shown their potential also in fish^49,59,63–65^. Members of *Gracilaria* genus have been investigated as infeed ingredients to improve health in fish, and despite some differences, overall, the inclusion of this seaweed biomass has a positive effect on fish health.

By using 16S rRNA amplicon sequencing we were able to identify more than 150 taxonomical features, and rarefaction indicated that sequencing was deep enough to identify all features in samples by reaching a plateau for all samples. Interestingly, although the total number of observed features was not different between the anterior and posterior intestine, we identified a different composition of the microbial communities from both compartments (Supplementary Fig. 1) based on the Bray–Curtis dissimilarities. Since differences in microbiome composition and functionality between fish intestinal compartments have been identified in other studies^60,66^, analyses were conducted separately for both sections. We observed that neither seaweed biomass nor its extract inclusion in diet changed community richness or diversity. These results differ from the reported by Rico et al.^67^, where higher richness of seabream intestinal communities was observed after being fed with diets including 15% *Gracilaria* or *Ulva* biomass. These differences can be attributed to the practiced inclusion rates. However, the modulatory effects of algae dietary inclusion in intestine microbial communities’ diversity have been in some cases contradictory. When considering macroalgae, no differences in diversity in seabream intestines were found by Abdala-Díaz et al.^68^ after 30 days of feeding with *Ulva rigida* at 25% inclusion. On the other hand, Tapia-Paniagua et al.^60^ found higher diversity in Senegalese sole anterior intestine microbiota after adding 5% *Ulva ohnoi* to the diet for 45 days. However, compared with other dietary supplements with potential as fish health modulators (e.g., probiotics, prebiotics), the algae effect on the gut has received limited attention with only a few published studies. Nevertheless, reported results allow us to infer that microbiome modulation by algae is highly dependent on host and algae species, inclusion rate, and duration of feeding among other factors, reinforcing the complexity of the gut microbial community and its relationships.

In terms of composition, *Proteobacteria*, *Firmicutes* and *Actinobacteria* have been reported as the most common phyla of intestinal bacteria in marine fish. The microbial community of seabass evaluated in this study was in line with the previously described in both intestine sections in any diet enrichment^13,23,67,69–71^. At the phylum level, the results showed that the inclusion of *Gracillaria gracilis* in the fish diet induced an evident change in the anterior intestine, decreasing Proteobacteria abundance, and this is in line with other studies that used *Ulva ohnoi*^60^. On the other hand, Actinobacteria abundance increased in the anterior intestine of fish fed with algae or algae extract. Other studies have shown different modulations with an unchanged or decreased abundance of this phylum and interestingly, the use of *Ulva ohnoi* or *Gracilaria* sp. increased Tenericutes and Firmicutes abundance, respectively^60,71^. The strong reduction of Vibrionales abundance observed in all groups with algae or algae extract is in line with other studies that showed less abundance of *Vibrio* and *Photobacterium* genera, both members of Vibrionales order, when fish were fed with algae supplemented diets^23,60,67^. Members of these genera are Gram-negative bacteria that gained notoriety partly due to the pathogenesis of some of its species. In particular, microorganisms from *Photobacterium* genus can be isolated from various marine surfaces and environments, including other organisms, with which they establish interactions that may be negative to the host^72^. *Photobacterium damselae*, is a fish pathogen with two subspecies, *P. damselae* subsp. *damselae* and *P. damselae* subsp. *piscicida* (*Phdp*) which is the infectious agent of pasteurellosis in fish. Due to its low specificity and high mortality rates, is liable for huge economic impacts in the industry on a global scale^28^. In the present study, the inclusion of *Gracilaria* as a functional feed additive influenced gut microbial composition, and one of the observed modulations was on the abundance of genus *Photobacterium.* In fish fed diets with *Gracilaria* inclusion, but not its extract, the relative abundance of *Photobacterium* was reduced, limiting its role as a permanent and latent member of the intestinal microbiota. This modulation was highlighted as the most relevant taxonomical modulation in the gut community by the random forest analysis, unravelling a probable relationship between in-feed administration of *Gracilaria* and *Photobacterium* species abundance. Although this requires further validation, this result is in line with the ones described by O’Sullivan et al.^73^, which suggested that some macroalgae polysaccharides (i.e., agar and carrageenan) may have inhibitory effects towards some microorganisms. When feeding European seabass with diets supplemented with 5% *Gracilaria gracilis* extract, Peixoto et al.^65^ found an increase in fish resistance to infection with *Photobacterium damselae* subsp. *piscicida*, which was explained by a higher antioxidant and immune response. Interestingly, Passos et al.^59^ also found an improved resistance against the same pathogen in gilthead seabream fed with diets similar to the ones used in this study. Since the overall physiological analysis did not indicate a direct cause of the resistance (i.e., immunostimulation), it was suggested gut microbiota could have played a role in this feature. A possible antagonistic relationship between the seaweed and this pathogenic bacterium was evaluated by Passos et al.^49^ with non-significant results. However, in this study we identified several clusters of correlations between some genera that indicate the bacteria relationships that are modulated by the algae-supplemented diet in both intestinal sections, but not by the algae extract.

In the anterior intestine, *Photobacterium* presented a possible antagonistic (i.e., negative correlation) relation with *Propionibacterium*, a microorganism that produces propionic acid, a short-chain fatty acid with antimicrobial properties^74^. There was an evident cluster where the genera *Burkholderia* and *Streptococcus* were correlated due to their abundance reduction in fish fed the diets with algae inclusion. Both genera include pathogenic species^75,76^ but for example, *Burkholderia* has also been linked to higher health performance in salmon^15,75^ whereas *Streptococcus* have been highlighted as probiotics in shrimp^77^. Another cluster of positively correlated genera was evidenced due to their higher abundance in fish fed the diet with 2.5% algae inclusion. The correlated genera, *Brachybacterium*, *Enterococcus*, *Stenotrophomonas*, *Brevibacterium*, *Dietzia* and *Bradyrhizobium* are naturally occurring taxa with different properties among them such as lipase production^78^, regulators of nitrogen and sulfur cycle^79^ or even opportunistic behavior^80,81^, and might be playing a relevant role in the modulation of the seabass intestinal microbiota community co-occurring in a well-orchestrated manner. Changes of these bacteria abundances and associations might have consequences for the microbiome functioning and its relationship with the host, in this case, promoting better health status and disease resistance^49,59^. While reporting the physiological assessment of fish from this trial, Passos et al.^49^ highlighted some histological alterations in the gut. Interestingly, in the groups where microbiota modulation was more noticed (ALGAE2.5 and ALGAE5) histological changes did not show a coherent pattern. When fish were fed ALGAE5 diet villus length were lower, whereas when fed ALGAE2.5 villus width increased. However, all algae or extract fed group had an increase in the number of goblet cells indicating an effect in the mucosa. Although in our study we found no significant correlation between microbial abundance and goblet cell quantification, this was probably due to limited sample number (i.e., replicates average was used since histology and microbial analysis were not obtained from same fish), and it should be targeted in a future study to understand the relation with microbial community.

Indeed, to deepen our knowledge on the extent of the observed microbial modulation, a microbiome functional prediction was performed and highlighted a possible modulation of several metabolic pathways in the microbiome of fish fed algae supplemented diet. For instance, an increase in heterolactic fermentation was predicted when including *Gracilaria* biomass in the diets, and this is probably linked with lactic acid bacteria (such as from the genus *Enterococcus*) activity and is likely to have beneficial outputs for the host intestinal health^82^. An increase in Bifidobacterium Shunt pathway was also predicted and it might be responsible for an increase of acetate and lactate in the gut lumen. These compounds acidify the intestinal lumen preventing the growth of harmful bacteria, and also serve as an energy source for intestinal epithelial cells^83^. Although the levels of these compounds were not assessed in this study, a further validation considering these assessments would confirm this possible beneficial effect *G. gracili*s dietary supplementation. Another interesting example is the ectoine biosynthesis pathway that was predicted to increase in the microbiome of fish fed algae supplemented diets. Ectoine is a natural compound found in higher concentrations in halophilic microorganisms and acts as a compatible solute for the survival of osmotic stress^84^. Its commercial form is issued in nutraceuticals as an enzyme stabilizer and cell protector for skin, and a similar role in the gut should be further investigated. It is worth mentioning that in the posterior intestine the modulation observed on the *Photobacterium* genus abundance was even more noticeable and here it was found with a positive correlation with members of the genera *Rubritalea* and *Gardnerella*, whereas the genera *Anaerobacillus*, *Micrococcus* and *Rubribacter* seem to have an antagonistic relationship with the pathogenic genus members. These interactions are yet to be studied and require further confirmation as well as the consequences for the microbiome functioning. However, it is worth mentioning that in the posterior intestine, it was predicted an in the pyruvate fermentation to acetate and lactate II and succinate fermentation to butanoate pathways in the microbiome of fish fed algae supplemented groups. Although these results are yet to be validated with target analysis, an increase in these pathways would lead to an increase in the production of short-chain fatty acids (e.g., butanoate) as well as acetate and lactate. If confirmed this will have beneficial consequences for the gut epithelium. More, understanding how dietary supplements modulate gut microbial interactions and their cross-talk with the host under different functional nutrition scenarios is of utmost importance nowadays. Correlating that information with fish physiological output will allow to move forward in fish intestinal health management in aquaculture.

In conclusion, supplementing seabass diets with *Gracilaria gracilis* biomass at 2.5% and 5% has an impact on gut microbiome composition. The diet did not alter the diversity and richness of the communities, however, alteration of the abundance patterns of some taxonomical groups was observed and at the genus level, several taxa presented correlation patterns that suggest a possible mutualistic/antagonistic coexistence. The modulation observed in the abundance of members of *Photobacterium* genus was the most relevant alteration exerted by the diets, with abundance reduction of the pathogen to undetectable levels.

## Methods

### Ethics statement

The current study was conducted complying with the ARRIVE guidelines and according to the European Directive 2010/63/EU. All experimental procedures were approved by DGAV (Portuguese Veterinary Authority) under the license 0421/000/000/2019 and by the Animal Welfare Committee of the Polytechnic Institute of Leiria.

### Algae collection, processing, and experimental diets

Algal biomass of *Gracilaria gracilis*, harvested from the Portuguese west coast was brought to Cetemares facility (MARE-Polytechnic of Leiria, Peniche, Portugal). All contaminants were removed and *G. gracilis* was thoroughly washed with seawater. Procedures were then followed to obtain dry algae powder and algal extract from the clean seaweed biomass. Algae powder was produced by drying the seaweed at 25 °C until constant weight and then grinding it to dust. The samples were stored at − 20 °C until use. The algal extract was prepared by drying the algal biomass at 25 °C until constant weight, grinding it into particles smaller than 200 µm, extracting twice with distilled water in a proportion of 1:10 (m:v) and then extracting in absolute ethanol in a proportion of 1:10 (m:v), all extractions were performed in a magnetic stirrer at room temperature for 30 min. The crude extract obtained was then filtered through a paper filter (Whatman nº4) and evaporated in a rotary evaporator at 40 °C. The extracts were frozen at − 20 °C until further processing. A specialized company (Sparos Olhão, Portugal) mixed the algal extract and powder in standard aquafeed^49^, considering an algal extract concentration of 0.35% (EXTRACT) and two algal powder concentrations, 2.5% (ALGAE2.5) and 5% (ALGAE5). Dry ingredients were mixed in a double-helix mixer (model RM90, Mainca, Barcelona, Spain) and ground (below 200 µm) in a micropulverizer hammer mill (model SH1, Hosokawa-Alpine, Augsburg, Germany). Subsequently, the oils were added to the mixtures, which were humidified with water and agglomerated by a low-shear and low-temperature extrusion process (Italplast West Heidelberg, VIC, Australia). Extruded pellets were dried in a vibrating fluid bed dryer (model DR100, TGC Extrusion, Roullet-Saint-Estèphe, France). Diets were packed in sealed plastic buckets and shipped to the experimental facilities (MARE-Polytechnic of Leiria, Peniche, Portugal) where they were stored at room temperature in a cool and aerated emplacement. Proximal composition of the diets was not different and was previously reported by Passos et al.^49^ and ash content ranged between 7.4 and 8.1%, and in relation to dry matter protein ranged between 52.2 and 53.5%, fat between 13.5 and 13.9% and energy between 21.7 and 21.9 (KJ g^−1^)/%DM.

### Fish and rearing conditions

The trial was performed at the Aquaculture Laboratory of MARE-Polytechnic Institute of Leiria (Peniche, Portugal) and al procedures were previously approved by the Ethical committee and were previously reported by Passos et al.^49^ for the first part of the study. European seabass (*Dicentrarchus labrax*) juveniles (17.49 ± 6.07 g; mean ± SD) were obtained from Estação Piloto de Piscicultura de Olhão (Instituto Português do Mar e da Atmosfera, I.P.) and were acclimated to the laboratory facilities for two weeks. After the quarantine period, fish were randomly distributed into 12 aquaria (60 L, 5.83 ± 0.31 kg m^−3^), connected to four recirculating aquaculture systems (RAS). The feeding trial lasted for 47 days, and water parameters such as temperature (19.93 ± 0.54 °C), salinity (31.53 ± 0.50), pH (8.48 ± 0.31) and O_2_ (87.87 ± 3.36%) (mean ± SD) were monitored daily. Water ammonium and nitrite levels in the tanks were kept below limits (< 0.05 mg L^−1^ and < 0.5 mg L^−1^, respectively). The fish from each treatment (4 diets and standard feed as control) were hand-fed, to apparent satiation, twice a day (9 a.m. and 4 p.m.). No mortality occurred during the experimental trial.

### Sampling procedure

By the end of the feeding period, 3 fish per replicate were sampled. The fish were anaesthetized with 2-phenoxyethanol (0.5 mL L^−1^) and euthanized by anaesthetic overdose and confirmation by severing the vertebral spine in the immediate post cranial region. Fish abdominal skin was washed with 70% ethanol and all the sampling procedures thereafter were performed under aseptic conditions. As the fish were put through fasting on the last day of the feeding period, the intestinal tracts were empty. Anterior and posterior intestine sections of 2 cm were collected separately, frozen in liquid nitrogen and stored at − 80 °C until processing.

### DNA extraction and 16S rRNA sequencing

Total genomic DNA was extracted from the anterior intestine and posterior intestine samples using a sterile scalpel to scrape internal contents and mucosa, applying the QIAamp Fast DNA Stool Kit (Qiagen, Hilden, Germany) according to the manufacturer’s instructions^12^. The integrity of isolated DNA was evaluated by agarose gel electrophoresis and DNA was quantified in a Qubit fluorometer (Thermofisher Scientific, Waltham, USA). Before library preparation, a PCR was performed to ensure the presence of bacterial DNA using universal primers for 16S rRNA (341F-5′-CCTACGGGNGGCWGCAG-3′ and 785R: 5′-GACTACHVGGGTATCTAATCC-3′)^85^. PCR reaction contained 1 × PCR buffer (DFS-Taq DNA polymerase, Bioron, Römerberg, Germany), 200 µM of dNTP’s mix, 0.5 µM of each primer and 1.5 µL of template DNA. PCR reaction was performed on a Biorad thermal cycler, with 5 min at 95 °C followed by 35 cycles of 4 min at 95 °C, 30 s at 60 °C and 50 s at 72 °C, and the final extension lasted 5 min at 72 °C. Only the samples with high DNA quality in both anterior and posterior intestine sections and with clear 16S rRNA amplicon bands (as observed by the agarose electrophoresis gel bands after PCR) were considered acceptable for the 16S rRNA amplicon sequencing (5 fish per treatment, in a total of 40 samples) were sent to STAB VIDA (Caparica, Portugal) for processing. Paired-end sequencing (2 × 300 bp read length) was performed from individual samples on a MiSeq system (Illumina) using the MiSeq Reagent Kit v3 according to the manufacturer’s instruction. Raw sequence data are available in the SRA database BioProject ID NCBI- PRJNA781597.

### Bioinformatic analysis and statistics

Sequences were demultiplexed by the sequencing provider inhouse software, and microbiome bioinformatics analysis was performed with QIIME 2 2020.8^86^. Paired-end raw sequences were filtered for quality, merged and chimeras removed by denoising with DADA2^87^. The obtained amplicon sequence variants (ASVs) were aligned with mafft^88^ and phylogeny was constructed with fasttree2^89^. Features appearing in only one sample were considered artefacts and were removed. Taxonomy was assigned to ASVs using the q2-feature-classifier^90^ using as database the Greengenes 13_8 99% OTUS reference sequences^91^. All sequences assigned for chloroplast or mitochondria were excluded and all results were separated by tissue (i.e., anterior and posterior intestine). For diversity analysis, samples were rarefied (i.e., randomly subsampled to the smallest library without replacement) to 29,959 sequences per sample. Here, alpha-diversity metrics (observed features, Chao1 and Shannon index) and beta-diversity metrics (Bray–Curtis dissimilarity), and Principal Coordinate Analysis (PCoA) were estimated using the q2-diversity script. Differences between the group’s alpha-diversity metrics were assessed using QIIME2 significance tests resulting in an evaluation with the Kruskal–Wallis test, whereas the group’s Bray–Curtis dissimilarities significance were tested by PERMANOVA which uses the dissimilarities between samples of the same group and compares them to the distances between groups^92,93^.

Differential abundance analysis was performed on non-rarefied data; however, a cumulative sum scaling (CSS) was performed to normalize data. This method accounts for heteroskedasticity of feature variance across values and controls the false discovery ratio in data^94,95^ therefore it is preferable to classical total sum scaling. A correlation network analysis was performed to identify possible interactions between microorganisms. Highlighting these interactions can provide valuable inputs on the microbiome function and if a specific diet promotes different interactions between taxa. SparCC^96^ correlation method was applied, considering 100 permutations, and retaining features with a correlation coefficient higher than 0.4 and respective P-value < 0.05. SparCC uses a log-ratio transformation and identifies taxa pairs different from background correlations by performing multiple iterations. Correlation analysis was performed based on FastSpar implementation available from the MicrobiomeAnalyst^97^. To identify microbial taxa that differentiate between groups the Random Forest algorithm was applied. This is a supervised machine-learning algorithm that identifies non-linear relationships by constructing multiple decision trees using a randomly selected subset of the data, allowing classification and selection of important features^98^. The random forest model was created using 500 trees.

Microbiome functional profiling prediction was performed with the Phylogenetic Investigation of Communities by Reconstruction of Unobserved States, named PICRUSt2^99^. This method is based on the idea that phylogenetically related organisms are more likely to have similar gene contents, and the algorithm uses several gene family databases. In this study, the functional profiles of the bacterial communities were predicted using the PICRUSt2 from the MetaCyc pathways database. The differences in functional profiling for the microbial communities in the anterior and posterior intestines of European seabass fed with different diets were characterized using Statistical Analysis of Metagenomics Profiles^100^. The significance level was always set to 0.05 considering a corrected P-value for false discovery ratio (FDR-corrected).

## Supplementary Information

Supplementary Figure 1.

Supplementary Figure 2.

Supplementary Table 1.

## Data Availability

The datasets generated and analyzed during the current study are available in the NCBI-SRA repository under the BioProject PRJNA781597.

## References

[1] David LA (2014). Diet rapidly and reproducibly alters the human gut microbiome. Nature.

[2] Butt RL, Volkoff H (2019). Gut microbiota and energy homeostasis in fish. Front. Endocrinol..

[3] Rinninella E (2019). What is the healthy gut microbiota composition? A changing ecosystem across age, environment, diet, and diseases. Microorganisms.

[4] Ni J, Yan Q, Yu Y, Zhang T (2014). Factors influencing the grass carp gut microbiome and its effect on metabolism. FEMS Microbiol. Ecol..

[5] Torrecillas S, Montero D, Izquierdo M (2014). Improved health and growth of fish fed mannan oligosaccharides: Potential mode of action. Fish Shellfish Immunol..

[6] Feng Q, Chen WD, Wang YD (2018). Gut microbiota: An integral moderator in health and disease. Front. Microbiol..

[7] Cordero H (2015). Modulation of immunity and gut microbiota after dietary administration of alginate encapsulated *Shewanella putrefaciens* Pdp11 to gilthead seabream (*Sparus aurata* L.). Fish Shellfish Immunol..

[8] Nayak SK (2010). Role of gastrointestinal microbiota in fish. Aquac. Res..

[9] Moriano-Gutierrez S, Ruby EG, McFall-Ngai MJ (2021). MicroRNA-mediated regulation of initial host responses in a symbiotic organ. mSystems.

[10] Clements KD, Pasch IBY, Moran D, Turner SJ (2007). *Clostridia* dominate 16S rRNA gene libraries prepared from the hindgut of temperate marine herbivorous fishes. Mar. Biol..

[11] Dehler CE, Secombes CJ, Martin SAM (2017). Environmental and physiological factors shape the gut microbiota of Atlantic salmon parr (*Salmo salar* L.). Aquaculture.

[12] Gonçalves AT, Gallardo-Escárate C (2017). Microbiome dynamic modulation through functional diets based on pre- and probiotics (mannan-oligosaccharides and *Saccharomyces cerevisiae*) in juvenile rainbow trout (*Oncorhynchus mykiss*). J. Appl. Microbiol..

[13] Serra CR, Oliva-Teles A, Enes P, Tavares F (2021). Gut microbiota dynamics in carnivorous European seabass (*Dicentrarchus labrax*) fed plant-based diets. Sci. Rep..

[14] Clements KD, Angert ER, Montgomery WL, Choat JH (2014). Intestinal microbiota in fishes: What’s known and what’s not. Mol. Ecol..

[15] Wang C, Sun G, Li S, Li X, Liu Y (2018). Intestinal microbiota of healthy and unhealthy Atlantic salmon *Salmo salar* L. in a recirculating aquaculture system. J. Oceanol. Limnol..

[16] Navarrete P, Espejo RT, Romero J (2009). Molecular analysis of microbiota along the digestive tract of juvenile Atlantic salmon (*Salmo salar* L.). Microb. Ecol..

[17] Ye L, Amberg J, Chapman D, Gaikowski M, Liu WT (2014). Fish gut microbiota analysis differentiates physiology and behavior of invasive Asian carp and indigenous American fish. ISME J..

[18] Molinari LM (2003). Bacterial microflora in the gastrointestinal tract of Nile tilapia, *Oreochromis niloticus*, cultured in a semi-intensive system. Acta Sci. Biol. Sci..

[19] Zhou Z (2009). Molecular characterization of the autochthonous microbiota in the gastrointestinal tract of adult yellow grouper (*Epinephelus awoara*) cultured in cages. Aquaculture.

[20] Yúfera M, Darías MJ (2007). Changes in the gastrointestinal pH from larvae to adult in Senegal sole (*Solea senegalensis*). Aquaculture.

[21] Macmillan JR, Santucci T (1990). Seasonal trends in intestinal bacterial flora of farm-raised channel catfish. J. Aquat. Anim. Health.

[22] Ma C, Chen C, Jia L, He X, Zhang B (2019). Comparison of the intestinal microbiota composition and function in healthy and diseased Yunlong Grouper. AMB Express.

[23] Egerton S, Culloty S, Whooley J, Stanton C, Ross RP (2018). The gut microbiota of marine fish. Front. Microbiol..

[24] Pérez T (2010). Host-microbiota interactions within the fish intestinal ecosystem. Mucosal Immunol..

[25] Verschuere L, Rombaut G, Sorgeloos P, Verstraete W (2000). Probiotic bacteria as biological control agents in aquaculture. Microbiol. Mol. Biol. Rev..

[26] Zrncic, S. Diagnostic manual for the main pathogens in European seabass and Gilthead seabream aquaculture. 75 (2020).

[27] Barnes AC, Dos Santos NMS, Ellis AE (2005). Update on bacterial vaccines: *Photobacterium damselae* subsp. *piscicida*. Dev. Biol..

[28] Toranzo AE, Magariños B, Romalde JL (2005). A review of the main bacterial fish diseases in mariculture systems. Aquaculture.

[29] Fernández Sánchez JL (2022). Assessing the economic impact of diseases in Mediterranean grow-out farms culturing European sea bass. Aquaculture.

[30] Essam HM, Abdellrazeq GS, Tayel SI, Torky HA, Fadel AH (2016). Pathogenesis of *Photobacterium damselae* subspecies infections in sea bass and sea bream. Microb. Pathog..

[31] Bakopoulos V (2003). Vaccination trials of sea bass, *Dicentrarchus labrax* (L.), against *Photobacterium damsela* subsp. *piscicida*, using novel vaccine mixtures. J. Fish Dis..

[32] Cabello FC (2006). Heavy use of prophylactic antibiotics in aquaculture: A growing problem for human and animal health and for the environment. Environ. Microbiol..

[33] Defoirdt T, Sorgeloos P, Bossier P (2011). Alternatives to antibiotics for the control of bacterial disease in aquaculture. Curr. Opin. Microbiol..

[34] Fu J (2017). Aquatic animals promote antibiotic resistance gene dissemination in water via conjugation: Role of different regions within the zebra fish intestinal tract, and impact on fish intestinal microbiota. Mol. Ecol..

[35] Carlson JM, Leonard AB, Hyde ER, Petrosino JF, Primm TP (2017). Microbiome disruption and recovery in the fish *Gambusia affinis* following exposure to broad-spectrum antibiotic. Infect. Drug Resist..

[36] Zhou L (2018). Environmental concentrations of antibiotics impair zebrafish gut health. Environ. Pollut..

[37] Carnevali O, Maradonna F, Gioacchini G (2017). Integrated control of fish metabolism, wellbeing and reproduction: The role of probiotic. Aquaculture.

[38] Hassaan MS, Soltan MA, Ghonemy MMR (2014). Effect of synbiotics between *Bacillus licheniformis* and yeast extract on growth, hematological and biochemical indices of the Nile tilapia (*Oreochromis niloticus*). Egypt. J. Aquat. Res..

[39] Dhanasiri AKS (2011). Changes in the intestinal microbiota of wild atlantic cod *Gadus morhua* L. upon captive rearing. Microb. Ecol..

[40] Holben WE, Williams P, Saarinen M, Särkilahti LK, Apajalahti JHA (2002). Phylogenetic analysis of intestinal microflora indicates a novel Mycoplasma phylotype in farmed and wild salmon. Microb. Ecol..

[41] Waagbø R, Remø SC (2020). Functional diets in fish health management. Aquac. Health Manag..

[42] Nagappan S (2021). Potential of microalgae as a sustainable feed ingredient for aquaculture. J. Biotechnol..

[43] Zerrifi SEA, Khalloufi FE, Oudra B, Vasconcelos V (2018). Seaweed bioactive compounds against pathogens and microalgae: Potential uses on pharmacology and harmful algae bloom control. Mar. Drugs.

[44] Mangott A (2020). *Ulva lactuca* as a functional ingredient and water bioremediator positively influences the hepatopancreas and water microbiota in the rearing of *Litopenaeus vannamei*. Algal Res..

[45] Francavilla M, Franchi M, Monteleone M, Caroppo C (2013). The red seaweed *Gracilaria gracilis* as a multi products source. Mar. Drugs.

[46] de Almeida CLF (2011). Bioactivities from marine algae of the genus *Gracilaria*. Int. J. Mol. Sci..

[47] Imjongjairak S (2016). Biochemical characteristics and antioxidant activity of crude and purified sulfated polysaccharides from *Gracilaria fisheri*. Biosci. Biotechnol. Biochem..

[48] Capillo G (2018). New insights into the culture method and antibacterial potential of *Gracilaria gracilis*. Mar. Drugs.

[49] Passos R (2021). Potential use of macroalgae *Gracilaria gracilis* in diets for European seabass (*Dicentrarchus labrax*): Health benefits from a sustainable source. Fish Shellfish Immunol..

[50] Silva-Brito F (2022). Dietary supplementation with Gracilaria gracilis by-products modulates the immune status and oxidative stress response of gilthead seabream (*Sparus aurata*) stimulated with *Photobacterium damselae* subsp. piscicida. Fish Shellfish Immunol..

[51] Afonso C, Correia AP, Freitas MV, Mouga T, Baptista T (2021). In vitro evaluation of the antibacterial and antioxidant activities of extracts of *Gracilaria gracilis* with a view into its potential use as an additive in fish feed. Appl. Sci..

[52] Praveen MA, Parvathy KRK, Balasubramanian P, Jayabalan R (2019). An overview of extraction and purification techniques of seaweed dietary fibers for immunomodulation on gut microbiota. Trends Food Sci. Technol..

[53] Azaza MS (2008). Growth of Nile tilapia (*Oreochromis niloticus* L.) fed with diets containing graded levels of green algae ulva meal (*Ulva rigida*) reared in geothermal waters of southern Tunisia. J. Appl. Ichthyol..

[54] Diler I, Tekinay AA, Güroy D, Güroy BK, Soyutürk M (2007). Effects of *Ulva rigida* on the growth, feed intake and body composition of common carp, *Cyprinus carpio* L. J. Biol. Sci..

[55] Vizcaíno AJ, Galafat A, Sáez MI, Martínez TF, Alarcón FJ (2020). Partial characterization of protease inhibitors of *Ulva ohnoi* and their effect on digestive proteases of marine fish. Mar. Drugs.

[56] Silva DM (2015). Evaluation of IMTA-produced seaweeds (Gracilaria, Porphyra, and Ulva) as dietary ingredients in Nile tilapia, *Oreochromis niloticus* L., juveniles. Effects on growth performance and gut histology. J. Appl. Phycol..

[57] Lyons PP, Turnbull JF, Dawson KA, Crumlish M (2017). Effects of low-level dietary microalgae supplementation on the distal intestinal microbiome of farmed rainbow trout *Oncorhynchus mykiss* (Walbaum). Aquac. Res..

[58] Norambuena F (2015). Algae in fish feed: Performances and fatty acid metabolism in juvenile Atlantic Salmon. PLoS ONE.

[59] Passos R (2021). Effect on health status and pathogen resistance of gilthead seabream (*Sparus aurata*) fed with diets supplemented with *Gracilaria gracilis*. Aquaculture.

[60] Tapia-Paniagua ST (2019). Modulation of intestinal microbiota in *Solea senegalensis* fed low dietary level of *Ulva ohnoi*. Front. Microbiol..

[61] Wan AHL, Davies SJ, Soler-Vila A, Fitzgerald R, Johnson MP (2019). Macroalgae as a sustainable aquafeed ingredient. Rev. Aquac..

[62] Murai U, Yamagishi K, Kishida R, Iso H (2021). Impact of seaweed intake on health. Eur. J. Clin. Nutr..

[63] Batista S (2020). Exploring the potential of seaweed *Gracilaria gracilis* and microalga *Nannochloropsis oceanica*, single or blended, as natural dietary ingredients for European seabass *Dicentrarchus labrax*. J. Appl. Phycol..

[64] Negm SS, Ismael NEM, Ahmed AI, El Asely AM, Naiel MAE (2021). The efficiency of dietary *Sargassum aquifolium* on the performance, innate immune responses, antioxidant activity, and intestinal microbiota of Nile Tilapia (*Oreochromis niloticus*) raised at high stocking density. J. Appl. Phycol..

[65] Peixoto MJ (2019). Protective effects of seaweed supplemented diet on antioxidant and immune responses in European seabass (*Dicentrarchus labrax*) subjected to bacterial infection. Sci. Rep..

[66] Gajardo K (2016). A high-resolution map of the gut microbiota in Atlantic salmon (*Salmo salar*): A basis for comparative gut microbial research. Sci. Rep..

[67] Rico RM (2016). Influence of the dietary inclusion of *Gracilaria cornea* and *Ulva rigida* on the biodiversity of the intestinal microbiota of *Sparus aurata* juveniles. Aquac. Int..

[68] Abdala-Díaz RT (2021). Effects of a short pulse administration of *Ulva rigida* on innate immune response and intestinal microbiota in *Sparus aurata* juveniles. Aquac. Res..

[69] Kim PS (2021). Host habitat is the major determinant of the gut microbiome of fish. Microbiome.

[70] Li Y (2021). Differential response of digesta- and mucosa-associated intestinal microbiota to dietary insect meal during the seawater phase of Atlantic salmon. Anim. Microbiome.

[71] Silva-Brito F (2021). Fish performance, intestinal bacterial community, digestive function and skin and fillet attributes during cold storage of gilthead seabream (*Sparus aurata*) fed diets supplemented with *Gracilaria* by-products. Aquaculture.

[72] Labella AM, Arahal DR, Castro D, Lemos ML, Borrego JJ (2017). Revisiting the genus *Photobacterium*: Taxonomy, ecology and pathogenesis. Int. Microbiol..

[73] O’Sullivan L (2010). Prebiotics from marine macroalgae for human and animal health applications. Mar. Drugs.

[74] Rossi B (2021). Antimicrobial power of organic acids and nature-identical compounds against two *Vibrio* spp.: An in vitro study. Microorganisms.

[75] Zhang Q-L (2019). The response of microbiota community to *Streptococcus agalactiae* infection in zebrafish intestine. Front. Microbiol..

[76] Mahboub HH (2022). Dietary black cumin (*Nigella sativa*) improved hemato-biochemical, oxidative stress, gene expression, and immunological response of Nile tilapia (*Oreochromis niloticus*) infected by *Burkholderia cepacia*. Aquac. Rep..

[77] Swain SM, Singh C, Arul V (2009). Inhibitory activity of probiotics *Streptococcus phocae* PI80 and *Enterococcus faecium* MC13 against Vibriosis in shrimp *Penaeus monodon*. World J. Microbiol. Biotechnol..

[78] Ringø E, Strøm E, Tabachek J-A (1995). Intestinal microflora of salmonids: A review. Aquac. Res..

[79] Ryan RP (2009). The versatility and adaptation of bacteria from the genus *Stenotrophomonas*. Nat. Rev. Microbiol..

[80] Abraham TJ, Paul P, Adikesavalu H, Patra A, Banerjee S (2016). *Stenotrophomonas maltophilia* as an opportunistic pathogen in cultured African catfish *Clarias gariepinus* (Burchell, 1822). Aquaculture.

[81] Koerner RJ, Goodfellow M, Jones AL (2009). The genus *Dietzia*: A new home for some known and emerging opportunist pathogens. FEMS Immunol. Med. Microbiol..

[82] Fijan S (2014). Microorganisms with claimed probiotic properties: An overview of recent literature. Int. J. Environ. Res. Public Health.

[83] Fushinobu S (2010). Unique sugar metabolic pathways of bifidobacteria. Biosci. Biotechnol. Biochem..

[84] Pastor JM (2010). Ectoines in cell stress protection: Uses and biotechnological production. Biotechnol. Adv..

[85] Thijs S (2017). Comparative evaluation of four bacteria-specific primer pairs for 16S rRNA gene surveys. Front. Microbiol..

[86] Bolyen E (2019). Reproducible, interactive, scalable and extensible microbiome data science using QIIME 2. Nat. Biotechnol..

[87] Callahan BJ (2016). DADA2: High-resolution sample inference from Illumina amplicon data. Nat. Methods.

[88] Katoh K (2002). MAFFT: A novel method for rapid multiple sequence alignment based on fast Fourier transform. Nucleic Acids Res..

[89] Price MN, Dehal PS, Arkin A (2010). Fasttree 2-approximately maximum-likelihood trees for large alignments. PLoS One.

[90] Bokulich NA (2018). Optimizing taxonomic classification of marker-gene amplicon sequences with QIIME 2’s q2-feature-classifier plugin. Microbiome.

[91] McDonald D, Price M, Goodrich J, Al E (2012). An improved Greengenes taxonomy with explicit ranks for ecological and evolutionary analyses of bacteria and archae. ISME J..

[92] Anderson, M. J. & Walsh, D. C. *Wiley StatsRef: Statistics Reference Online* (Wiley, 2014). 10.1002/9781118445112.

[93] Anderson, M. J. Permutational multivariate analysis of variance (PERMANOVA). In *Wiley StatsRef: Statistics Reference Online* 1–15 (Wiley, 2017). 10.1002/9781118445112.stat07841.

[94] Pereira M, Wallroth M, Jonsson V, Kristiansson E (2018). Comparison of normalization methods for the analysis of metagenomic gene abundance data. BMC Genom..

[95] Dillies M (2013). A comprehensive evaluation of normalization methods for Illumina high-throughput RNA sequencing data analysis. Bioinform.

[96] Friedman J, Alm EJ (2012). Inferring correlation networks from genomic survey data. PLoS Comput. Biol.

[97] Chong J, Liu P, Zhou G, Xia J (2020). Using Microbiome Analyst for comprehensive statistical, functional, and meta-analysis of microbiome data. Nat. Protoc..

[98] Knights D, Costello E, Knight R (2011). Supervised classification of human microbiota. FEMS Microbiol. Rev.

[99] Douglas GM (2020). PICRUSt2 for prediction of metagenome functions. Nat. Biotechnol..

[100] Parks D, Tyson G, Hugenholtz P, Beiko RG (2014). STAMP: Statistical analysis of taxonomic and functional profiles. Bioinformatics.

