# Supplementary figures and images for: Modulatory effect of Gracilaria gracilis on European seabass gut microbiota community and its functionality
Source: Sci Rep. 2022 Sep 1;12:14836. doi: 10.1038/s41598-022-17891-9 (PMC9437047; doi:10.1038/s41598-022-17891-9)

Supplementary Figure 1

Unweighted unifrac PCoA


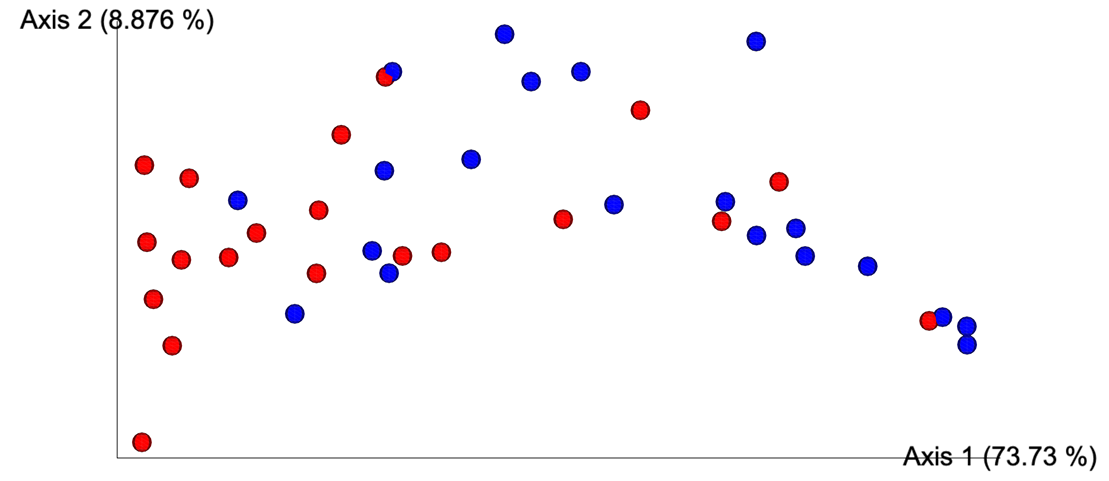


Weighted unifrac PCoA


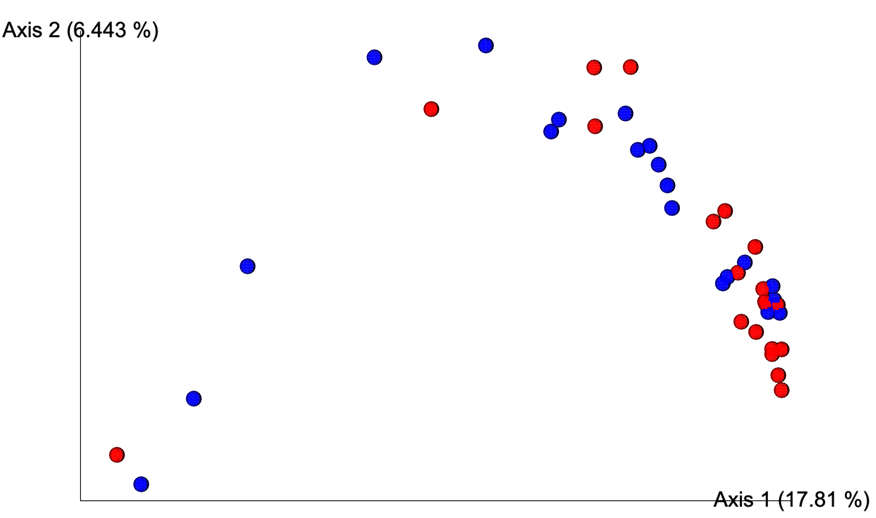


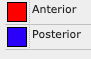

Supplement: Supplementary file 1 — Supplementary Figure 1. [file 41598_2022_17891_MOESM1_ESM.docx]
