# Supplementary material for: Modulatory effect of Gracilaria gracilis on European seabass gut microbiota community and its functionality
Source: Sci Rep. 2022 Sep 1;12:14836. doi: 10.1038/s41598-022-17891-9 (PMC9437047; doi:10.1038/s41598-022-17891-9)

**Supplementary Figure 2**. Heat map with hierarchical clustering of metagenome based on the microbiome predicted metabolic pathways modulated by diet in anterior (A) and posterior (B) intestine. Sample clustering was performed considering the control group (CTRL), the groups with algae inclusion (Algae) and the group with seaweed extract inclusion (Extract).


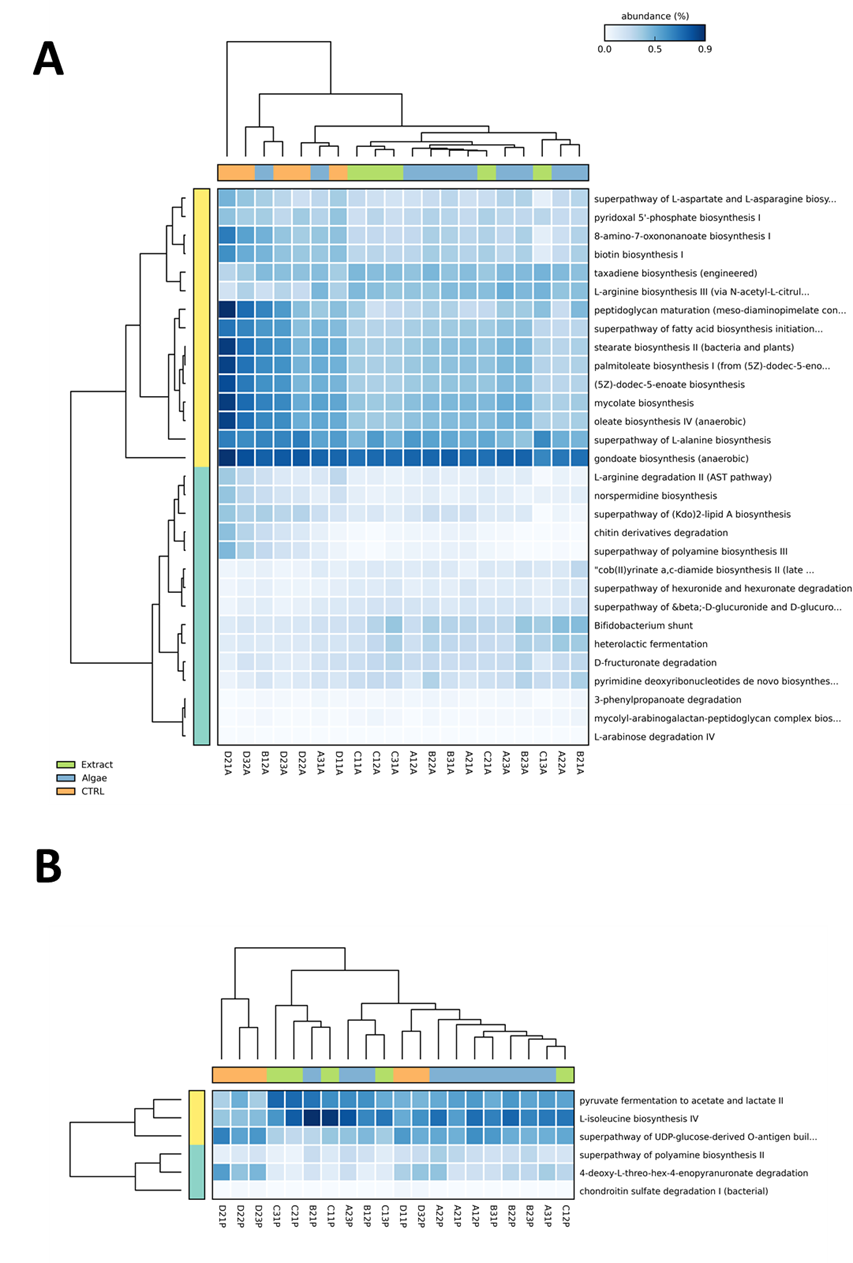

Supplement: Supplementary file 2 — Supplementary Figure 2. [file 41598_2022_17891_MOESM2_ESM.docx]
